# Treatment with zolpidem after ethanol administration potentiates the expression of ethanol-induced behavioral sensitization in mice

**DOI:** 10.1590/1414-431X202010034

**Published:** 2020-06-26

**Authors:** N.R.N. Brandão, M. Libarino-Santos, E.A.V. Marinho, T.S. Oliveira, A.L.N. Borges, A.P. Oliveira, D. Oliveira-Campos, N. Azevedo-Souza, V.F.L. Santos, L.F. Berro, A.J. Oliveira-Lima

**Affiliations:** 1 Departamento de Ciências da Saúde, Universidade Estadual de Santa Cruz, Ilhéus, BA, Brasil; 2 Department of Psychiatry and Human Behavior, University of Mississippi Medical Center, Jackson, MS, USA

**Keywords:** Ethanol, Behavioral sensitization, Zolpidem, Open-field, Mice

## Abstract

Contradictory findings suggest that the behavioral and abuse-related effects of ethanol are mediated by its action at α1 subunit-containing GABA_A_ (α1GABA_A_) receptors. In the present study, we investigated the effects of a sub-chronic post-ethanol administration treatment with zolpidem, an α1-preferring positive allosteric modulator at GABA_A_ receptors, on the subsequent expression of ethanol-induced behavioral sensitization in mice. Animals received ethanol (1.8 g/kg, *ip*) or saline treatments every other day for 15 days (8 treatment sessions) and were subsequently treated with zolpidem (0.5 mg/kg, *ip*) or vehicle 4 times on alternate days. At the end of the treatment phase, animals were challenged with saline or ethanol on separate days for the evaluation of the expression of conditioned locomotion and behavioral sensitization. Eight-day treatment with ethanol did not lead to the development of ethanol-induced behavioral sensitization. Animals treated with ethanol and subsequently administered vehicle showed similar locomotion frequencies during the last ethanol challenge compared to the control group receiving ethanol for the first time. Animals treated with ethanol and subsequently administered zolpidem expressed behavioral sensitization to ethanol during the ethanol challenge. The present study adds to the literature by providing further evidence of a role of α1GABA_A_ receptors on the behavioral effects of ethanol. Because of the current highly prevalent co-abuse of ethanol and benzodiazepine drugs in humans, the use of zolpidem and other α1GABA_A_ receptor ligands during ethanol withdrawal should be monitored carefully.

## Introduction

Ethanol use disorder is a devastating disease, resulting in a series of organic, psychological, social, and economic problems. In the United States alone, nearly 17 million people suffer from ethanol-related disorders, being responsible for 88,000 deaths and costing more than $223.5 billion annually (1). Ethanol is frequently consumed in combination with other drugs of abuse, and the combination of ethanol and sedative-hypnotic drugs, such as zolpidem, has been associated with increased likelihood of being admitted to intensive care units (2,3).

Zolpidem and ethanol share similar mechanisms of action. Ethanol is a central nervous system depressant, and acts by potentiating γ-aminobutyric acid (GABA)ergic neurotransmission via action at the GABA_A_ receptor (4). Zolpidem is an α1-preferring positive allosteric modulator at GABA_A_ receptors (5). Although several subtypes of GABA_A_ receptors have been implicated in the abuse-related effects of ethanol (6), the role of α1-containing GABA_A_ (α1GABA_A_) receptors on ethanol abuse remains controversial. Previous studies in rodents have shown a reduced preference for ethanol in α1GABA_A_ null mutant mice (7) and reduced ethanol self-administration after treatment with α1GABA_A_ receptor antagonists in rats (8). However, self-administration studies in non-human primates have shown little evidence of a contribution of α1GABA_A_ receptors on the reinforcing effects of ethanol (9), and subject-rated reinforcing effects of zolpidem were not increased by ethanol in humans (10).

In order to further elucidate the role of α1GABA_A_ receptors on ethanol-induced behaviors, the present study was designed to investigate the effects of a sub-chronic post-ethanol administration treatment with zolpidem on the subsequent expression of ethanol-induced behavioral sensitization in mice.

## Material and Methods

### Subjects

Three-month-old Swiss male mice (LaBIO, Universidade Estadual de Santa Cruz, UESC) were used in the experiments. Animals were housed 10 per cage under controlled ventilation, temperature (22−23°C), and lighting conditions (12 h light/dark, lights on at 6:30 am) with free access to water and food. The experiments were in accordance with the National Institute of Health Guide for Care and Use of Laboratory Animals (8th edition, revised 2011) and the Brazilian Law No. 11,794, and were approved by the Institutional Ethical Committee of UESC.

### Drugs

Zolpidem (0.5 mg/kg) (Pfizer^®^, USA) was dissolved in 1% Tween 80 and subsequently diluted in 0.9% saline. Ethanol (1.8 g/kg) (Merck^®^, USA) was diluted in 0.9% saline. All solutions were administered intraperitoneally (*ip*) at a volume of 10 mL/kg.

### Open-field evaluation

Locomotor activity was measured in the open-field apparatus. The apparatus consisted of a circular wooden arena (40 cm in diameter and 50 cm high) with an open top and a floor divided into 19 approximately similar regions delimited by three concentric circles intersected by radial line segments. Animals were exposed to the open-field individually during 10-min sessions. Locomotor activity was tracked using the ANY-maze software (version 5.1, Stoelting, USA) and a webcam was suspended overhead.

### Experimental design

**Figure 1:**
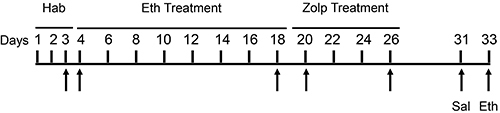
Experimental design. Hab: habituation. Eth treatment: *ip* injection every other day of saline (Sal) or ethanol (Eth, 1.8 g/kg). Zolp treatment: *ip* injection every other day of vehicle or zolpidem (Zolp, 0.5 mg/kg) in the open-field apparatus. Sal challenge: *ip* injection of saline. Eth challenge: *ip* injection of ethanol (1.8 g/kg). Arrows indicate days in which behavior was quantified.

Figure 1 illustrates the experimental design. Forty male mice were given a 10-min habituation session in the open-field for 3 consecutive days after a saline injection, and locomotor activity was quantified on day 3. Animals were then allocated into 2 groups (N=20 per group). The behavioral sensitization protocol (“ethanol treatment” phase) was conducted according to a protocol previously used by our research group (11). Animals received treatments of either ethanol (Eth) or saline (Sal) every other day for 15 days (8 treatment sessions, days 4 to 18). Five minutes after injections, animals were individually placed in the open-field for 10 min. Locomotor activity (distance traveled during the session) was measured on the 1st and 15th days of this phase.

Forty-eight hours after the last ethanol session, the zolpidem treatment phase began. Mice received an *ip* injection of vehicle (Veh) or zolpidem (Zol) every other day for 7 days (4 sessions, days 20 to 26), forming the following groups: Sal-Veh, Eth-Veh, Sal-Zol, and Eth-Zol (N=10 per group). Five minutes after each injection, mice were individually placed in the open-field for 10 min. Locomotor activity was measured on the 1st and last days of this phase.

Four days after the last treatment injection (day 31), all animals were acutely challenged with saline (*ip*) to evaluate conditioned responses in the open-field apparatus. Forty-eight hours after the saline challenge (day 33), animals were tested for drug-induced expression of behavioral sensitization to ethanol. All animals received an injection of 1.8 g/kg ethanol and were individually placed in the open-field apparatus.

### Statistical analysis

Before conducting the parametric tests, all variables were checked for normality (Shapiro-Wilk test) and homogeneity (Levene's test), which validated the use of the parametric test. Data were analyzed by one- or two-way analysis of variance (ANOVA), with or without repeated measures, or Student's *t*-test for paired samples (within-group comparisons). *Post hoc* comparisons were performed using Duncan's *post hoc* test. A P value less than 0.05 was considered to be a statistically significant difference.

## Results

**Figure 2:**
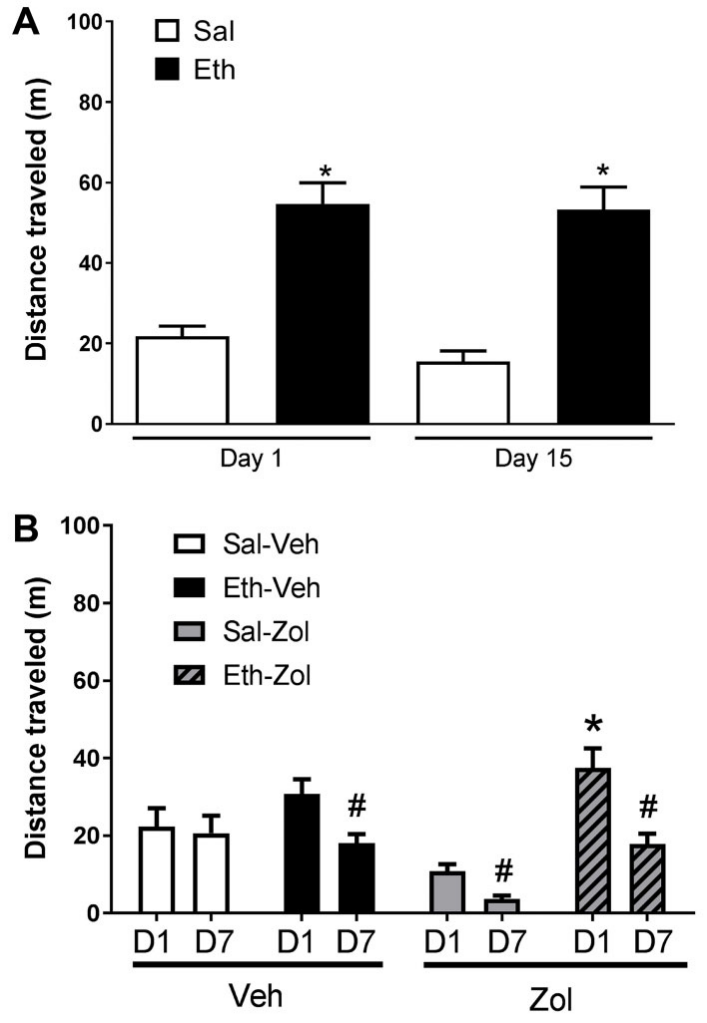
Locomotor activity quantification in the open-field apparatus demonstrating (**A**) acute hyperlocomotion induced by ethanol (Eth, 1.8 g/kg) (Day 1) and ethanol-induced behavioral sensitization (Day 15) after a 15-day intermittent treatment (8 ethanol injections) and (**B**) the behavioral effects of *ip* treatment with either zolpidem (Zol, 0.5 mg/kg) or vehicle (Veh) during the post-sensitization phase for 7 intermittent days (D1 to D7). Data are reported as means±SE. *P<0.05 compared to the respective control group (A: Sal, B: Sal-Zol); ^#^P<0.05 compared to itself on D1 (paired sample Student's *t*-test).

Analysis of the 3rd habituation session revealed no significant difference between groups (data not shown). During the ethanol treatment phase, one-way repeated measures ANOVA revealed a significant effect of treatment (Sal *vs* Eth) (F(1,38)=67.38, P<0.0001), but not time (Day 1 *vs* Day 15) (F(1,38)=0.84, P>0.05) or interaction between treatment and time (F(1,38)=0.36, P>0.05). *Post hoc* comparisons indicated that ethanol induced hyperlocomotion (Eth > Sal, Day 1), an effect that was not sensitized after repeated ethanol administration (Day 1=Day 15) (Figure 2A).

During the zolpidem treatment phase, two-way repeated measures ANOVA revealed a significant effect of time (Day 1 *vs* Day 7) (F(1,72)=17.2, P<0.0001), ethanol treatment (Sal *vs* Eth) (F(1,72)=22.07, P<0.0001), and zolpidem treatment (Veh vs Zol) (F(1,72)=4.93, P<0.05), as well as a significant interaction between time and ethanol treatment (F(1,72)=5.54, P<0.05) and ethanol treatment and zolpidem treatment (F(1,72)=12.43, P<0.001).

*Post hoc* comparisons showed that acute treatment with zolpidem in animals treated with saline, but not ethanol, induced hypolocomotion in mice (Sal-Zol < Sal-Veh, Day 1). Zolpidem-induced hypolocomotion was sensitized after repeated administration (Sal-Zol Day 7 < Sal-Zol Day 1). Although ethanol-treated animals did not express conditioned locomotion to ethanol on the 1st day of the zolpidem treatment phase (Eth-Veh=Sal-Veh), the distance traveled was significantly decreased over time in ethanol-treated animals, indicating an extinction effect (Figure 2B).

**Figure 3:**
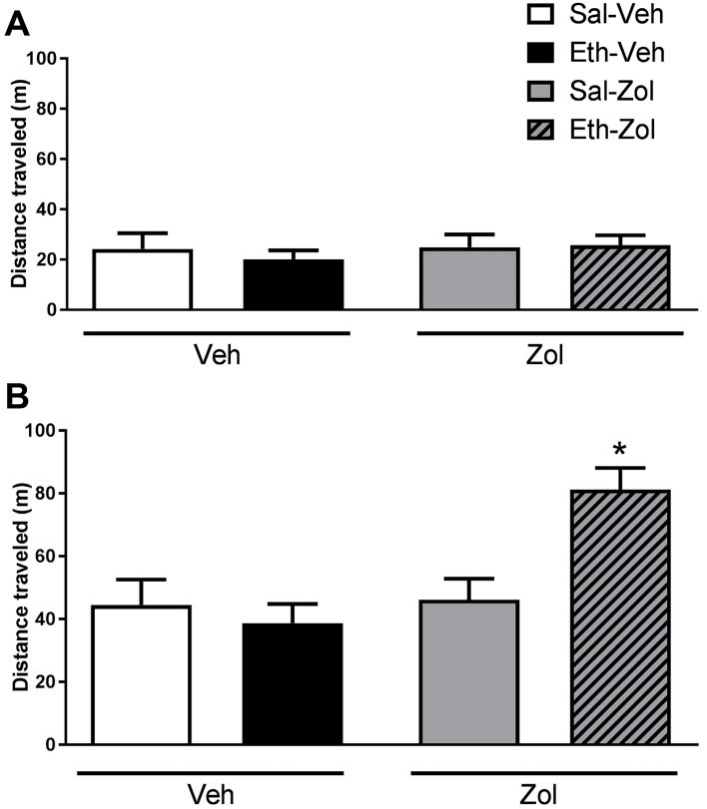
Locomotor activity quantification in the open-field during the (**A**) saline (Sal) and (**B**) ethanol (Eth) challenges after ethanol and/or zolpidem treatments. Data are reported as means±SE. *P<0.05 compared to the respective control group (B: Sal-Zol) (ANOVA).

During the saline challenge, two-way repeated measures ANOVA revealed no significant effect of ethanol treatment (F(1,9)=0.5, P>0.05), zolpidem treatment (F(1,9)=0.15, P>0.05), or interaction between ethanol and zolpidem treatments (F(1,72)=0.3, P>0.05) (Figure 3A). Regarding the ethanol challenge, two-way repeated measures ANOVA revealed a significant interaction effect between ethanol treatment and zolpidem treatment (F(1,9)=10.42, P<0.05). *Post hoc* analysis showed that animals sensitized to ethanol and subsequently treated with zolpidem, but not vehicle, expressed behavioral sensitization to ethanol, with animals in the Eth-Zol group having traveled longer distances than animals in all other groups (Figure 3B).

The two-way repeated measures ANOVA considering ethanol and zolpidem treatments as factors and habituation, Days 1 and 15 of ethanol treatment, Days 1 and 7 of zolpidem treatment, and saline and ethanol challenges as repeated measures revealed individual effects of time (F(6,252)=23.34, P<0.0001) and ethanol treatment (F(1,252)=64.25, P<0.0001), as well as interactions between time and ethanol treatment (F(6,252)=8.615, P<0.0001), time and zolpidem treatment (F(6,252)=4.456, P<0.001), ethanol treatment and zolpidem treatment (F(1,252)=7.934, P<0.01), and time *vs* ethanol treatment *vs* zolpidem treatment (F(6,252)=2.528, P<0.05).

*Post hoc* multiple comparisons showed that the locomotor activity of the Eth-Veh group did not change across ethanol treatment/challenge days (Day 1 *vs* Day 15 *vs* ethanol challenge), indicating that this group indeed did not express locomotor activity during the ethanol challenge. On the other hand, the Eth-Zol group did show a significantly higher locomotor frequency during the ethanol challenge compared to itself during Days 1 and 15 of the ethanol treatment phase (P=0.02 and P=0.0007, respectively).

## Discussion

In the present study, treatment with zolpidem after sub-chronic administration of ethanol (sensitization phase) promoted the expression of ethanol-induced behavioral sensitization. While animals in the Eth-Veh group exhibited similar locomotion frequency compared to control animals receiving ethanol for the first time (Sal-Veh), animals previously sensitized with ethanol and treated with zolpidem expressed higher locomotion frequencies than all other groups. Of note, animals receiving ethanol for the first time (Sal-Veh and Sal-Zol groups) showed similar locomotor activity levels during the ethanol challenge, suggesting that zolpidem did not simply potentiate the acute locomotor effects of ethanol and, instead, promoted the expression of ethanol-induced behavioral sensitization.

Zolpidem-induced cross sensitization was only evident during the ethanol challenge after a prolonged drug-free period. According to Lessov and Phillips (12), repeated ethanol administration associated with the test apparatus can promote modifications in neural pathways that mediate locomotor activity so that these pathways become more sensitive and responsive to a subsequent ethanol challenge. In fact, previous studies from our group have also shown that a drug-free interval is necessary for the expression of ethanol-induced behavioral sensitization (13). These neuroadaptations would explain the expression of behavioral sensitization after a drug-free period following a sensitization protocol and zolpidem treatment, and suggest that zolpidem might be modulating or further contributing to ethanol-induced neuroadaptations.

Ethanol interacts with several neurotransmitter systems (14,15), and ethanol-induced activation of the mesolimbic dopaminergic pathway seems to be the main mechanism underlying acute ethanol-induced locomotor stimulation (16,17). The ventral tegmental area (VTA) is one of the major regions in this pathway, and is predominantly comprised of dopamine neurons (∼70%) and GABA interneurons (∼20%) (18,19). Of note, ethanol-induced firing of dopamine neurons in the VTA seems to be modulated by ethanol-induced decreased firing of VTA GABAergic interneurons (20).

GABA is the main inhibitory neurotransmitter of the central nervous system, with presynaptic and post-synaptic action at inotropic (GABA_A_) and metabotropic (GABA_B_) receptors. Each GABA_A_ receptor is composed of five subunits, and the final composition of each receptor determines its distinct physiological and pharmacological properties (21). GABAergic interneurons located in the VTA selectively express α1-containing GABA_A_ receptors in rats (22,23). Thus, studies have focused on the investigation of the role of α1GABA_A_ receptors on ethanol-induced behaviors, with contradictory findings. While rodent studies show favorable evidence for a potential role of α1GABA_A_ receptors on ethanol preference (7) and ethanol drinking (8), non-human primate studies show little to no evidence of a contribution of this receptor to the abuse-related effects of ethanol (9).

The present study adds to the literature by providing further evidence of a role of α1GABA_A_ receptors in the behavioral effects of ethanol in rodents. More specifically, while 8 intermittent treatments with ethanol alone did not lead to the expression of behavioral sensitization in mice, animals treated with ethanol and subsequently administered zolpidem expressed ethanol-induced behavioral sensitization under our experimental conditions. These findings suggest that zolpidem extended the effects of ethanol when administered in the open-field apparatus. It is important to note, however, that treatment with zolpidem induced hypolocomotion in mice, indicating that the dose of zolpidem used in the present study might have induced a sedative effect in mice, opposed to the stimulant dose of ethanol. However, in the absence of zolpidem, when animals in the Zol- *vs* Veh-treated groups no longer showed differences in locomotor activity (saline challenge), the long-lasting effects of sub-chronic treatment with zolpidem after ethanol sensitization became evident. Our findings are in agreement with a previous study demonstrating that a 7-day treatment with zolpidem led to the development of mesolimbic dopamine-dependent neural plasticity in mice (24). It is important to note, however, that a previous study did not observe locomotor-enhancing effects of zolpidem in animals sensitized with ethanol. In the study by Linsenbardt and Boehm (25), the authors use a strain of mice known to be particularly susceptible to the development of ethanol-induced behavioral sensitization. In contrast to the present study, the authors did observe the development of behavioral sensitization to ethanol within the 15-day sensitization period. After the ethanol sensitization period, zolpidem did not potentiate the locomotor activity of mice. These data suggest that the cross-sensitization observed in the present study was, perhaps, only present because animals did not express sensitization to ethanol during the sensitization protocol in the first place.

Studies on the interaction between GABA_A_ receptor ligands and the abuse-related effects of ethanol become extremely relevant in light of the current highly prevalent co-abuse of ethanol and benzodiazepine drugs in humans (26). Because behavioral sensitization is thought to share neuroadaptations with drug craving in humans (27), data from the present study suggest that the use of zolpidem during ethanol withdrawal, such as in the context of hypnotic drug use due to Eth withdrawal-induced sleep impairment (28,29), may favor the installation of compulsive behavior.

Our findings suggested that α1GABA_A_ receptors play an important role on ethanol-induced behaviors, and that the use of zolpidem and other GABA_A_ receptor ligands in the context of ethanol abuse should be monitored carefully.
